# Around the Hospitals

**Published:** 1893-11-18

**Authors:** 


### AROUND THE HOSPITALS.

NOTICE TO THE MANAGERS OF INSTITUTIONS.—Special space is now reserved for the insertion of notices of hospital meetings and festivals. The Editor requests that all notices may reach THE HOSPITAL Office, 428, Strand, at least one week before the date of each meeting.

**The Manchester Southern Hospital.**—The pressure on the accommodation of this hospital for women and children continues, and the intended new institution will not be provided before it is needed. Already the site has been obtained. It is situated in the Oxford Road and Stanley Grove. Through the generosity of the council of Owens College, the freehold has been handed over to the authorities of the hospital. Towards the new buildings £6,000 has already been subscribed, but this is only half the sum needed. During the past year 500 women and children have been received, and 1,500 maternity cases attended. The finances are not very satisfactory, and the accounts show a balance of £397 to the bad. It is to be hoped that the acquisition of new buildings will enable the authorities to procure more funds, and thus carry on a useful work under less difficulties.

**The Derby Royal Infirmary.**—The annual meeting of this institution is a feature in the locality. The importance placed upon the event serves to enhance the interest taken in it both in the town and in the neighbourhood. The eighty-fourth anniversary meeting recently held proved no exception to the rule, the Bishop of Southwell preaching the customary sermon in All Saints' Church. The meeting was well attended, and great enthusiasm and interest shown. Although the Derby Infirmary is in a tentative condition, awaiting the opening of the new institution, which it is hoped will take place in May, the work of healing is carried on with unabated vigour. The number of in-patients show a decided increase on those of the previous year, and this is especially noticeable in the ophthalmic department. The administration has undergone several changes, including the election of a new Matron, Miss Carvosso, in place of Miss Oakely resigned, and the Secretary, Mr. Carnt, now resides in the institution. This last arrangement has proved an excellent one financially for the institution, £100 having been saved in gas, coal, and water during the past year. Wherever such items come under the control of a conscientious and responsible officer the change in expenditure is always marked, and in the commissariat departments of all institutions the greatest economy is effected where the direct supervision of the higher officials possessing the requisite knowledge, obtains. The expenditure of the infirmary amounted to £5,623. The income provided for this sum, and for the furnishing of the new nurses' home, which occasion was the principal event of the hospital year. The new infirmary will certainly entail a larger expenditure, and for this reason the President, Mr. G. H. Strutt, M.P., appealed to the governors at the annual meeting to increase their subscriptions. At this meeting Mr. Walter Evans' munificent intention to build a third block to the new infirmary at a cost of £12,000 was announced. In this and other ways the inhabitants of Derby and its neighbourhood show their interest and approval of the scheme for a new institution, not the least gratifying mark of approbation being shown in the increased contributions from the working classes.

